# Supplementary material for: Direct experimental observation of blue-light-induced conformational change and intermolecular interactions of cryptochrome
Source: Commun Biol. 2022 Oct 18;5:1103. doi: 10.1038/s42003-022-04054-9 (PMC9579160; doi:10.1038/s42003-022-04054-9)
Supplement: Supplementary file 2 — Supplementary Information [file 42003_2022_4054_MOESM2_ESM.pdf]

Supplementary Information

Direct Experimental Observation of Blue-Light-Induced Conformational Change and Intermolecular Interactions of Cryptochrome

Pei Li[1,†], Huaqiang Cheng[1,†], Vikash Kumar[2], Cecylia Severin Lupala[2], Xuanxuan Li[2], Yingchen Shi[2], Chongjun Ma[1], Keehyoung Joo[3], Jooyoung Lee[4], Haiguang Liu[2,5,*], Yan-Wen Tan[1,*]

[1]*State Key Laboratory of Surface Physics, Department of Physics, Fudan University, Shanghai, 200433, China*
[2]*Beijing Computational Science Research Center, Beijing 100193, China*
[3]*Center for Advanced Computation, Korea Institute for Advanced Study, Seoul, 02455, Republic of Korea*
[4]*School of Computational Sciences, Korea Institute for Advanced Study, Seoul, 02455, Republic of Korea*
[5]*Physics Department, Beijing Normal University, Haidian, Beijing 100875, People's Republic of China*

[†]*These authors contributed equally to this work.*

**Corresponding to: ywtan@fudan.edu.cn; hgliu@csrc.ac.cn*

This supplementary information includes:

Supplementary Tables

Table S1 Summary of protein production and purification conditions.

Table S2 The statistics of smFRET fitting results.

Table S3 The statistics of single-molecule interaction assay.

Table S4 The statistics of single-molecule dimerization assay.

Table S5 The statistics of single-molecule interaction and dimerization assay.

Table S6 Summary of the SEC-SAXS experiments and analysis results.

Supplementary Figures

Figure S1 Photoreduction of purified wild-type *Cra*CRY.

Figure S2 Schematic diagram of single molecule experiment design and *Cra*CRY monomer confirmed by bleaching step screening in dark conditions.

Figure S3 SEC curves obtained by SEC experiments alone and combined SEC-SAXS experiments.

Figure S4 The series analysis plots from BioXTAS RAW.

Figure S5 Predicted secondary structure for CTE domain and SAXS-driven MD simulation results.
Figure S6 The comparison of monomeric *Cra*CRY SAXS profiles under dark and lit conditions.
Figure S7 Interaction between ROC15(GARP) and FRET construct revealed by GST pull-down and the GST control experiments.
Figure S8 Blue light dependent dimerization and interactions between ROC15(GARP) and *Cra*CRY with BSA added.
Figure S9 Circadian cycles in protein expression levels for *Cra*CRY and ROC15 and the lifetime distributions of *Cra*CRY:ROC15 double overexpressing control.
Figure S10 SAXS data and the protein models for dimeric PHR/*Cra*CRY or protein complexes.
Figure S11 Blue light dependent dimerization, photoreduction and interaction of *Cra*CRY with TCEP or DTT.
Figure S12 Measurement of Förster Radius (R_0) of FRET Dye Pair Atto 550 /Atto 647 in *Cra*CRY buffer.
Figure S13 SDS-PAGE images of *Cra*CRY(wt) and FRET-construct during protein expression and purification process.
Figure S14 The constructs of ROC15(GARP) and PHR domain used in the methods.

Supplementary Movies

Movie S1 Refinement of *Cra*CRY protein by SAXS-driven MD simulations (dark).
Movie S2 Refinement of *Cra*CRY protein by SAXS-driven MD simulations (lit).
Movie S3 Refinement of *Cra*CRY protein by SAXS-driven MD simulations (trRosetta-lit).
Movie S4 *Cra*CRY protein dynamics with conventional equilibrium MD simulations.

Supplementary References

Table S1 Summary of protein production and purification conditions.

Sample	Plasmid				Expression			Purification
	Vector	Resist.	Cloning Sites	Tag	Temp.	IPTG (μM)	Medium	
*Cra*CRY (wt)	pET 28(a)	Kana	SacI/ HindIII	N-His	20°C 16 h	200	LB	His affinity column + SP column
*Cra*CRY PHR								
*Cra*CRY CTE	pET 52(b)	Amp	kpnI/ SalI	N-Strep -His	16°C 16 h	200	LB	SP column
FRET construct	pET 52(b)	Amp	kpnI/ SalI	N-Strep -His	16°C 12 h	200	LB	His affinity column + Mono Q column
*Cra*CRY::mCitrine	pET 52(b)	Amp	kpnI/ SalI	N-Strep -His	20°C 20 h	250	TB	SP column
ROC15(GARP) ::mCherry	pGEX	Amp	BamHI/ XhoI	N-GST -His	20°C 20 h	250	TB	His affinity column + Mono Q column

* All of the *Cra*CRY proteins were produced in *E.coli* BL21(DE3) cells in dark, with 20 μM free FAD in medium.

** Before testing, a Superdex 200 10/300 GL column (GE) had been employed to further purify all the proteins.

Table S2 The statistics of smFRET fitting results.

Sample	molecules	Data points	Fitting results
Monomer Dark state	13	2649	50 ± 13 Å
Monomer Lit state	22	2869	65 ± 12 Å
Dimer Lit state	65	22591	48 ± 12 Å

* Values following the "±" symbol are the confidence intervals (95%).

Table S3 The statistics of single-molecule interaction assay.

Conditions		Group 1	Group 2	Group 3	Group 4	Total	
						Fraction	Molecules
Dark	Fraction	6.9%	11.5%	2.2%	5.3%	6.5 ± 2.7%	996
	Molecules	58	116	507	319		
Lit	Fraction	13.5%	20.6%	40.9%	18.2%	23.3 ± 8.8%	1930
	Molecules	96	68	1250	516		

* In single-molecule pull-down assays, only the number of photobleaching steps was defined and counted for statistics.

** Values following the "±" symbol are the statistical standard deviation, using as the error bars in Fig.3e.

Table S4 The statistics of single-molecule dimerization assay.

Conditions		Group 1	Group 2	Group 3	Group 4	Group 5	Total	
							Fraction	Molecules
Dark	Fraction	11.4%	10.3%	3.5%	6.5%	5.7%	7.5 ± 2.7%	1430
	Molecules	273	116	198	31	812		
Lit	Fraction	30.9%	34.2%	21.9%	21.7%	16.9%	25.1 ± 5.9%	1505
	Molecules	233	225	314	83	650		

* Data have been divided into different groups based on the experiment dates.

** Values following the "±" symbol are the statistical standard deviation, using as the error bars in Fig.4c.

Table S5 The statistics of single-molecule interaction and dimerization assay.

**Conditions**	**Dimer**				**Interaction**			
	**without ROC15**		**with ROC15**		**among Monomers**		**among Dimers**	
	**Fraction**	**Molecules**	**Fraction**	**Molecules**	**Fraction**	**Molecules**	**Fraction**	**Molecules**
**Dark**	6.1 ± 2.1%	951	1.5 ± 2.2%	45	6.8 ± 2.9%	925	1.6 ± 2.3%	71
**Lit**	15.5 ± 5.1%	1298	2.2 ± 3.0%	554	25.1 ± 8.8%	1610	10.4 ± 11.8%	314

* Data have been divided into different groups based on the experiment dates.

** The errors marked in red are larger than the fraction values, while implies the appearance of the components with both interaction and dimerization happening may be overestimated from the sensitive single-molecule statistics.

Table S6 Summary of the SEC-SAXS experiments and analysis results

(a) Sample details

	***Cra*Cry(full)**	***Cra*Cry (PHR)**	**GST-GARP**
Organism	*C. reinhardtii*		
Source (Catalogue No. or reference)	*E.coli* expressed		
UniProt sequence ID (residues in construct)	A8J8W0	A8J8W0(1-489)	B1B5J3(377-445)
Extinction coefficient ε (wavelength and units) (A280, M-1 cm-1)	107830	106340	49850
Molecular mass M from chemical composition (KDa)	64.9	55.4	33.5
Concentration (range/values) measured and method	2-5mg/ml	1-3mg/ml	1-3mg/ml
Solvent composition and source	150 m*M* NaCl, 50 m*M* Hepes pH 7.4, 5% glycerol		

(b) SAXS data collection parameters

Source, instrument and description or reference	Ref. J. Appl. Cryst. (2016) 49, p1428-1432
Wavelength (Å)	0.9184
Beam geometry (size, sample-to-detector distance)	340μm x 60μm (H x V), 2.30 m
q-measurement range (Å-1)	0.01-0.47
Absolute scaling method	normalized to transmitted intensity recorded in ion chamber post sample
Basis for normalization to constant counts	Take silver behenate as standard to set the mask, then normalize the 2D images
Method for monitoring radiation damage, X-ray dose where relevant	SAXS data were collected as sample was continuously flowing through the cross section; multiple measurements were compared to assess the radiation damage
Exposure time, number of exposures	1 s per frame, until sample fully flows through the SEC. signals are averaged over 20 frames centered around the SEC peak
Sample temperature (ºC)	25

(c) Software employed for SAS data reduction, analysis and interpretation

SAS data reduction	*BioXTAS RAW*
Basic analyses: Guinier, P(r)	*PRIMUS* (*ATSAS 2.8.0*; Franke et al., 2017)
volume (e.g. Porod volume VP)	PRIMUS (ATSAS 2.8.0; Franke et al., 2017)
Shape/bead modelling	decodeSAXS (He et al., 2020)
Molecular graphics	UCSF *Chimera*

(d) Structural parameters

	***Cra*Cry (dark)**	***Cra*Cry (lit, monomer)**	***Cra*Cry (lit, dimer)**	***Cra*Cry (PHR,dark)**	***Cra*Cry (PHR,lit,monomer)**	***Cra*Cry (PHR,lit,dimer)**	***Cra*Cry+GST-GARP**
**Guinier Analysis**							
I(0) (cm-1)	50.56±0.16	108.61± 0.45	22.23±0.25	36.21±0.11	29.95± 0.12	6.46± 0.13	21.07±0.19
Rg (Å)	31.67 ±0.80	31.83±2.7	47.24±1.70	24.98±0.30	24.35±0.34	37.36±1.86	47.33±1.51
q-range (Å-1)	0.022~0.043	0.02~0.04	0.01~0.026	0.026 ~ 0.052	0.02~0.053	0.01~0.034	0.012~0.027
Quality-of-fit parameter (with definition)	0.69	0.85	0.92	0.87	0.93	0.89	0.98
**P(r) analysis**							
I(0) (cm-1)	51.29	111.2	21.75	36.46	30.34	6.06	19.3
Rg (Å)	33.54	34.11	46.31	25.37	25.05	36.63	43.4
dmax (Å)	123.17	130.91	141.03	83.71	81.83	108.32	134.67
q-range (Å-1)	0.021-0.251	0.022~0.252	0.009-0.17	0.028~0.216	0.02~0.25	0.025~0.213	0.02 ~0.25
Quality-of-fit parameter (with definition)	0.72	0.72	0.91	0.94	0.93	0.65	0.84
Porod Volume (Å^3)	93100	89700	356000	72000	71800	135000	184000

(e) Atomistic modelling

	***Cra*Cry (dark)**	***Cra*Cry (lit, monomer)**	***Cra*Cry (lit, dimer)**	***Cra*Cry (PHR,dark)**	***Cra*Cry (PHR,lit,monomer)**	***Cra*Cry (PHR,lit,dimer)**	***Cra*Cry+GST-GARP**
Method	Prediction + SAXS-MD	Prediction + SAXS-MD	fitting to cryoEM model	crystal structure	crystal structure	fitting to cryoEM model	Prediction + ZDOCK
q-range for fitting	0.021-0.251	0.022~0.252	0.009-0.17	0.028~0.216	0.02~0.25	0.025~0.213	0.02 ~0.25
Symmetry assumptions	n/a	n/a	n/a	n/a	n/a	n/a	n/a
χ value	1.13	1.13	1.06	0.98	1.14	0.96	1.13
Adjustable parameters in the model fit				solvent layer density contrast			
Domain/subunit coordinates and contacts, regions of				5ZM0	5ZM0		

Figure S1 Photoreduction of purified wild-type *Cra*CRY.

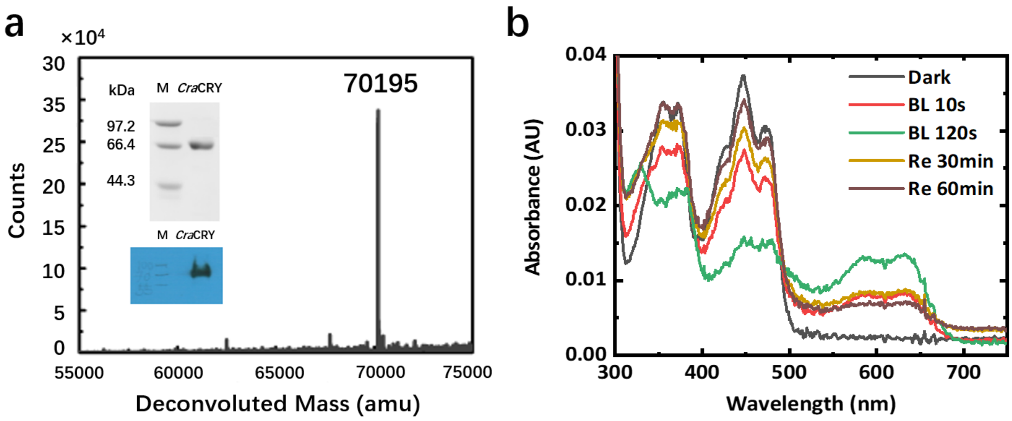

Fig. S1 Photoreduction of purified wild-type *Cra*CRY. a. ESI mass spectrum assay of wild-type *Cra*CRY. ESI determined molecular weight is 70195 (theoretical value 70195.7) daltons. The upper inset is the SDS-PAGE showing the purity of our sample， and the lower inset shows the result of western blot using antibody for His tag in the wild-type *Cra*CRY. These assays demonstrate that the *Cra*CRY had been expressed and purified successfully. **b.** Photoreduction of purified *Cra*CRY. These absorption spectrums were monitored by UV-VIS spectrometer. Before blue-light illumination, the *Cra*CRY proteins are in FAD oxidized state (black line, Dark state). With 2 min blue-light illumination, the protein will be partially reduced to FADH state (green line, BL 120 s). This process is reversible. The reduced protein will return to FAD oxidized state after remaining under dark conditions for 60 min.

Figure S2 Schematic diagram of single molecule experiment design and *Cra*CRY monomer confirmed by bleaching step screening in dark conditions.

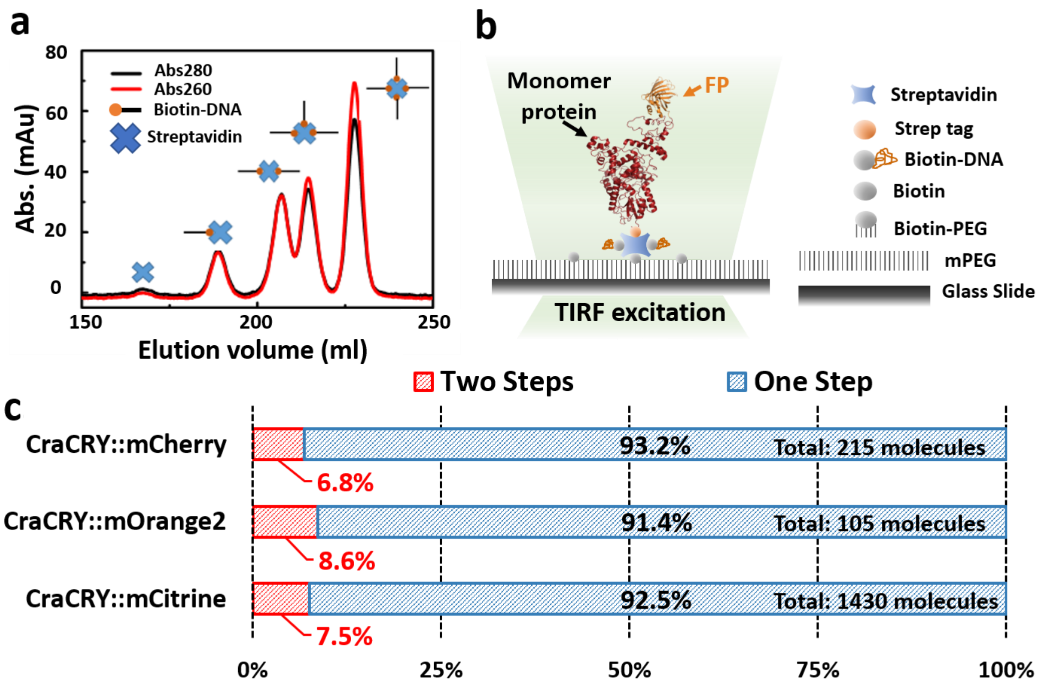

Fig. S2 Schematic diagram of single molecule experiment design and *Cra*CRY monomer confirmed by bleaching step screening in dark conditions. a. The trajectory of bivalent streptavidin eluted from Mono Q column. The different peaks correspond to different number of sites in one streptavidin (Amresco) molecule blocked by biotin-PC-DNAs (Tsingke Biotechnology)[1]. The proteins in the third highest peak around 208 mL, containing two biotin-PC-DNAs per streptavidin molecule, are needed for ensuring the single immobilization site per streptavidin molecule. **b.** The schematics of single molecule immobilized on the passivated slide. In single molecule experiment, the glass slide has been passivated by the mixture of mPEG-SCM (5k, Biomatrik) and Biotin-PEG-SCM (6k, Biomatrik)[2]. Biotin-PEG is used to bind one site of the bivalent streptavidin on the slide. The target protein fused with the strep tag, can been tethered to the other site of the bivalent streptavidin. This design can ensure that at most one protein can be linked to a streptavidin molecule through the interaction between streptavidin and strep tag. Please refer to the literature for more details[1,2]. **c.** The monomer (blue) / dimer (red) fractions of *Cra*CRY::mCherry, *Cra*CRY::mOrange2 and *Cra*CRY::mCitrine under dark conditions.

Figure S3 SEC curves obtained by SEC experiments alone and combined SEC-SAXS experiments.

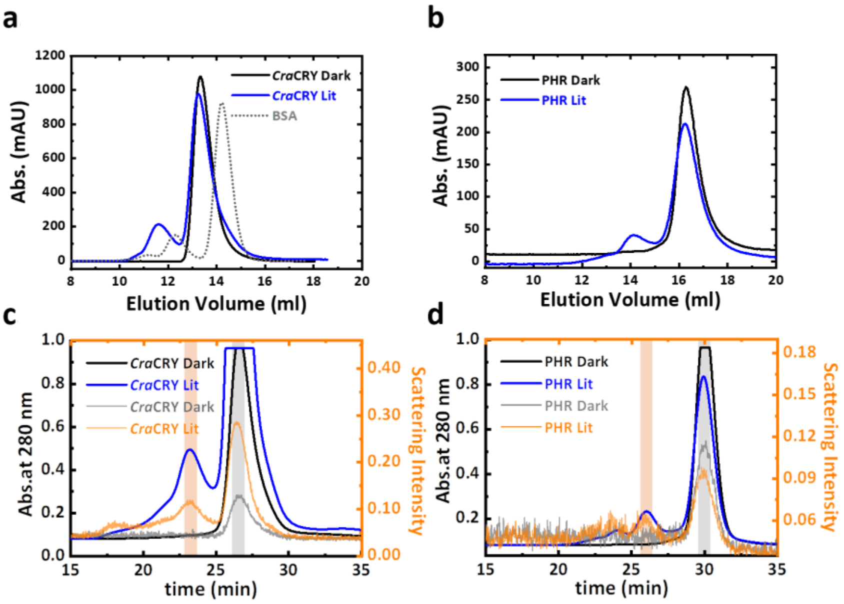

Fig. S3 Blue light dependent dimerization of *Cra*CRY and PHR domain. a. SEC curves of *Cra*CRY under dark and lit conditions. The dotted line is the calibration curve from the mixture of monomeric and dimeric BSA proteins, of which the molecular weight is about 66 kDa for monomeric BSA, very close to *Cra*CRY(wt) (70 kDa). For *Cra*CRY, only one peak appears at 13.6 mL under dark condition (black line), while two peaks at 11.8 mL and 13.2 mL appear under lit condition (blue line). The extra peak at 11.8 mL corresponds to the dimer of *Cra*CRY. **b.** SEC curves of PHR domain under dark and lit conditions. Similar to *Cra*CRY, compared with dark curve (black line), the extra peak in lit curve (blue line) shows the PHR domain can also be dimerized upon blue-light illumination. SEC was performed at room temperature with Superdex 200 (GE). The flow rate is 0.8–1.0 ml·min^{-1}. **c.** SEC and scattering intensity curves of *Cra*CRY under dark and lit conditions obtained by SEC-SAXS experiments. **d.** SEC and scattering intensity curves of PHR domain under dark and lit conditions obtained by SEC-SAXS experiments. In SEC-SAXS experiments, the elution times were recorded instead of elution volumes and the absorption signals (at 280 nm, black lines under dark conditions and blue lines under lit conditions) were saturated since the protein concentrations were very high. The scattering intensities of *Cra*CRY and PHR domain under dark (grey line) and lit (orange line) conditions are also shown respectively. And the regions marked by grey and orange blocks correspond to the samples used for SAXS analysis. The grey regions were for SAXS analysis on monomers, while the orange regions correspond to dimers.

Figure S4 The series analysis plot for *Cra*CRY SEC-SAXS experiments.

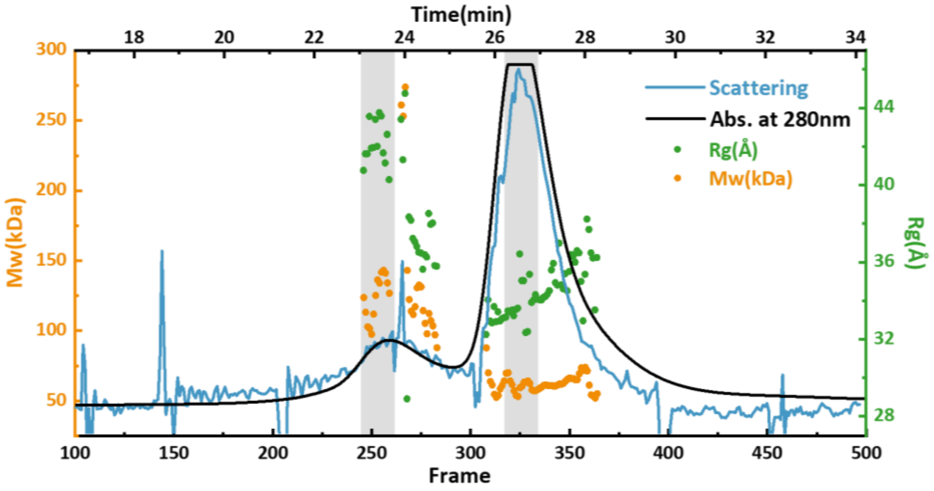

Fig. S4 The series analysis plot for *Cra*CRY SEC-SAXS experiment. The X-axes correspond to the flow-through time in SEC assay (top) and the frame number during SEC-SAXS experiment (bottom). The black and blue solid lines represent the absorption of sample at 280 nm and the integrated X-ray scattering intensities, respectively. The platform of absorption trace near 26-27 min appeared because the threshold of detector was exceeded. The gray area near 250 frame is the signal of dimeric *Cra*CRY and near 325 frame is that of monomeric *Cra*CRY. Green and orange dots exhibited the estimated Radius of gyration (Rg) and molecular weight (Mw) values for both monomeric and dimeric *Cra*CRY proteins.

Figure S5 Predicted secondary structure for CTE domain and SAXS-driven MD simulation results.

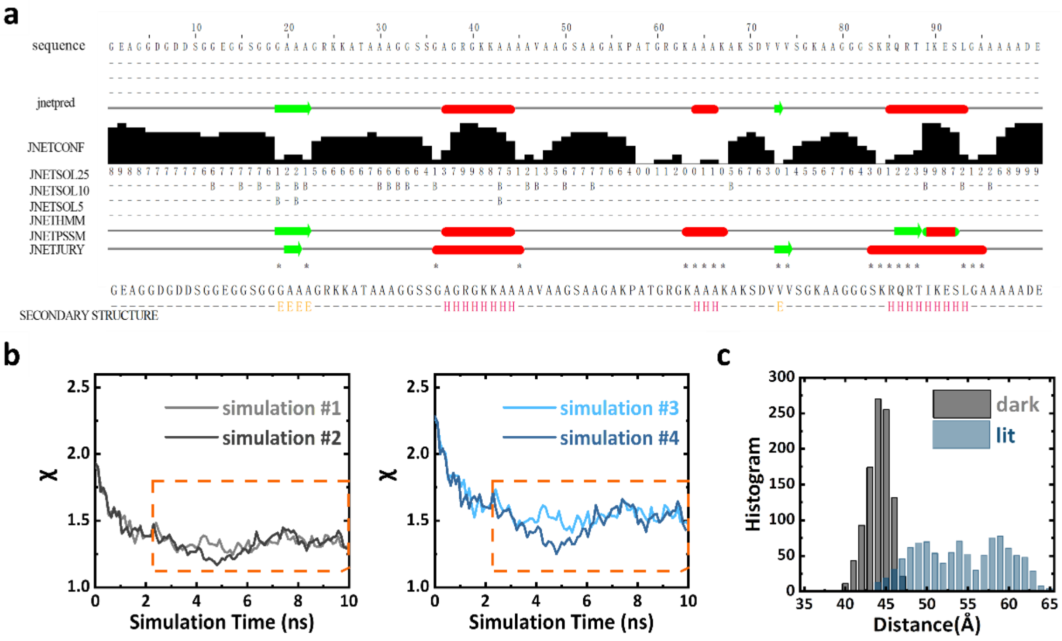

Fig. S5 Predicted secondary structure for CTE domain and SAXS-driven MD simulation results. a. Prediction result for CTE from the Jpred server, revealing helix and beta-strand regions in disordered- structure dominated CTE domain. **b.** The progress of model refinement against SAXS data in SAXS-driven MD simulations (see supplementary Movies S1,S2). The chi-score calculated by least squared fitting to experimental data decreased within the first 2 ns, indicating improvement in model-data consistency. Chi-score for structures sampled in two simulation trajectories were calculated for both dark and lit SAXS data fitting. The fitting to the dark state SAXS data is shown on the left sub-panel, and the lit cases are on the right sub-panel. The structure ensembles from two trajectories exhibit similar goodness-of-fit to SAXS data, but their conformations are better distinguished by the CTE orientations (see Figure 2 in main text). **c.** The histograms of distance between the two labelled residues (Cα atoms) for smFRET experiments. The refined structure ensembles were denoted as dark and lit, according to the distance features observed in smFRET experiment under the corresponding conditions.

Figure S6 The comparison of monomeric *Cra*CRY SAXS profiles under dark and lit conditions.

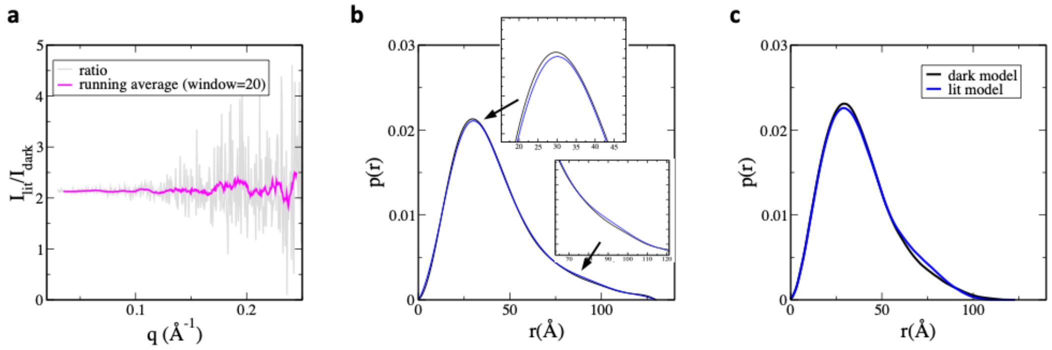

Fig. S6 The comparison of monomeric *Cra*CRY SAXS profiles under dark and lit conditions. a. The ratio between lit and dark SAXS profiles. The gray color shows the ratio at each q value, and the purple lines indicate the running average with a window size of 20 data points. **b.** The distance distribution p(r) calculated from the dark and lit SAXS profiles, using the same Dmax (130 Å). The p(r) of lit state (blue) shows slight expansion compared to the dark state (black), mainly manifested in the regions displayed as insets. **c.** The p(r) calculated from atomic models obtained from SAXS-driven MD simulations. A similar expansion trend is recovered in the atomic models. Note that the differences between (c) and (b) are due to the solvent layer, which is not considered in the atomic models but nonetheless contributes to the SAXS profiles measured in experiments.

Figure S7 Interaction between ROC15(GARP) and FRET construct revealed by GST pull-down and the GST control experiments.

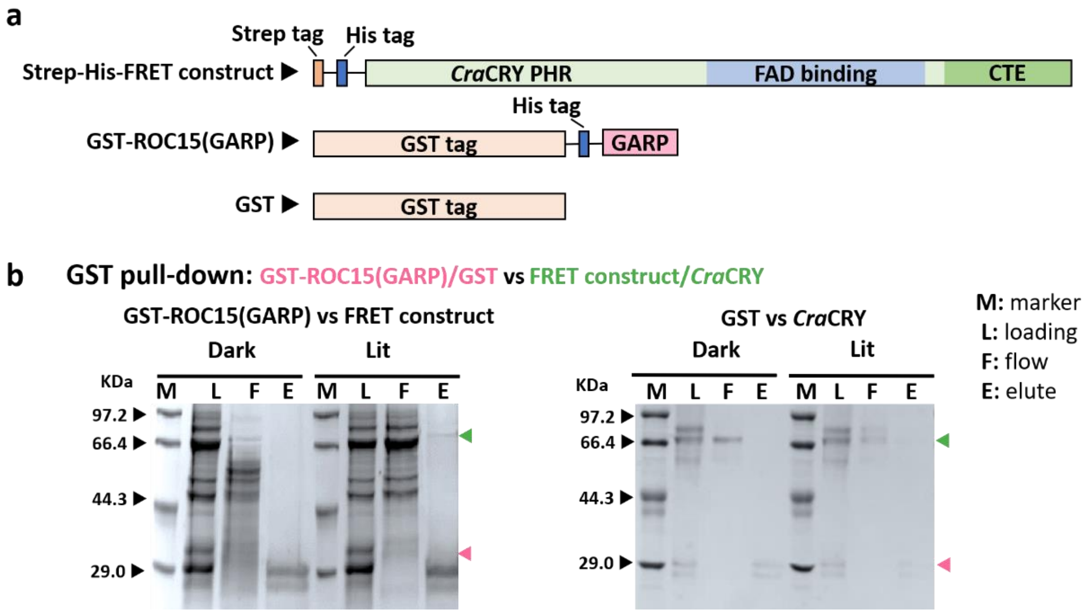

Fig. S7 Interaction between ROC15(GARP) and FRET construct revealed by GST pull-down and the GST control experiments. a. Sketch map of protein designs for ROC15(GARP) and FRET construct by GST pull-down and the control experiment. **b.** Protein-protein interaction complex pull-down displayed by SDS-PAGE. Green arrows mark the band of FRET construct (left panel) or the *Cra*CRY(wt) (right panel). Pink arrows mark the band of GST-ROC15(GARP) (left) or GST (right). The left image shows that both ROC15(GARP) and FRET construct can also be eluted from the GST column in blue light dependent manner, showing that the mutation does not eliminate the interaction between ROC15(GARP) and *Cra*CRY. This observation also shows that FRET construct preserves the signal transduction function. Either dark or lit, the right image shows there is no interaction between GST and *Cra*CRY (wt), demonstrating that the *Cra*CRY/PHR/FRET construct can only be baited by ROC15 (GARP), not the GST tag.

Figure S8 Blue light dependent dimerization and interactions between ROC15(GARP) and *Cra*CRY with BSA added.

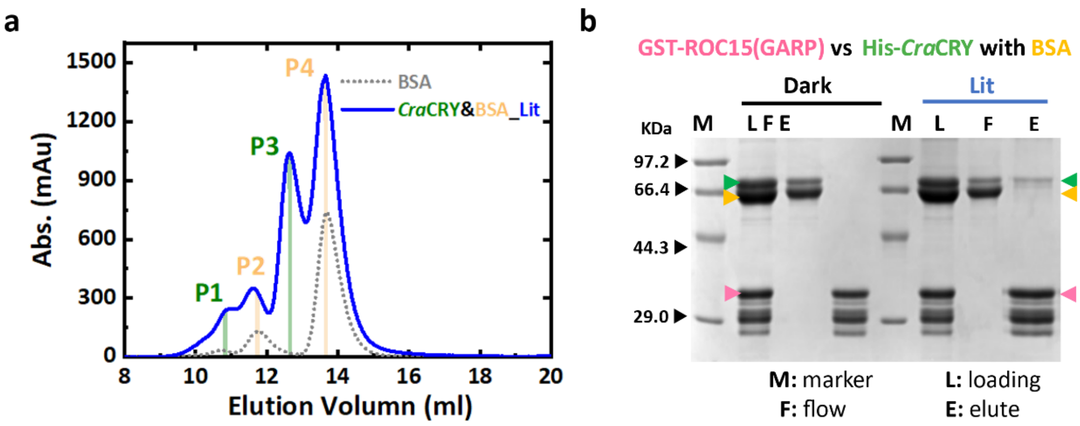

Fig. S8 Blue light dependent dimerization and interactions between ROC15(GARP) and *Cra*CRY with BSA added. a. SEC curves of BSA only (grey dots) and *Cra*CRY with BSA added (blue solid) upon blue-light illumination. P1 and P3 represent the positions of dimeric and monomeric *Cra*CRY (green vertical lines), respectively. While, P2 and P4 represent the positions of dimeric- and monomeric- BSA (Yellow vertical lines), respectively. **b.** Pull-down molecular complex analyzed using SDS-PAGE. Green arrows mark the *Cra*CRY(wt). Pink arrows mark the band of GST-ROC15(GARP). Yellow arrows mark the BSA. In the loading sample, the mass concentration ratio of *Cra*CRY, GST-GARP and BSA is 1:1:1.2. These images show that both the blue light dependent dimerization and interactions between ROC15(GARP) and *Cra*CRY are specific and will not be altered in the presence of BSA.

Figure S9 Circadian cycles in protein expression levels for *Cra*CRY and ROC15 and the lifetime distributions of *Cra*CRY:ROC15 double overexpressing control.

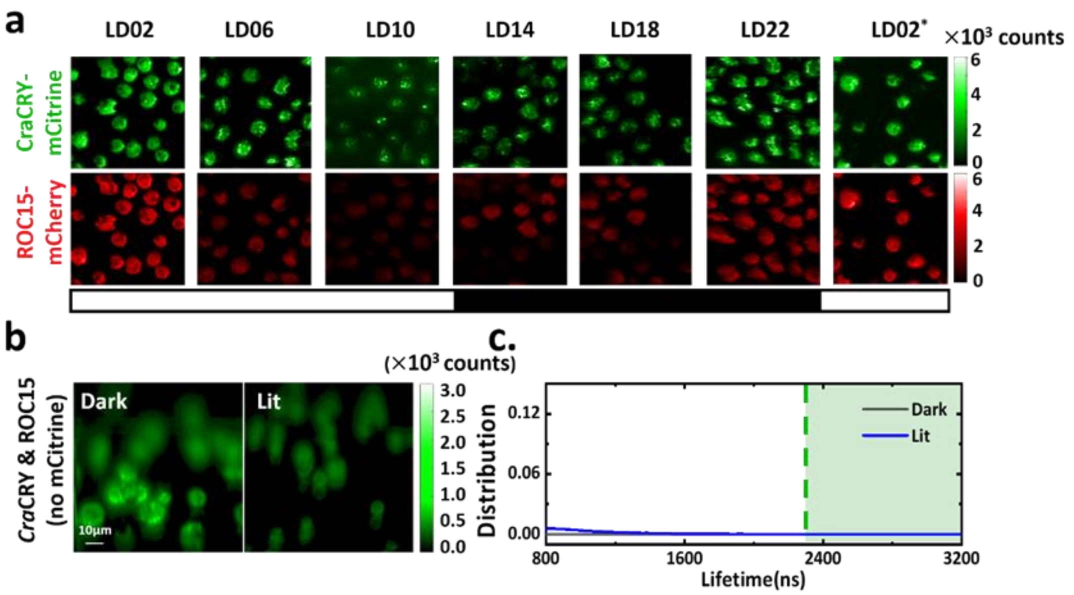

Fig. S9 Circadian cycles in protein expression levels for *Cra*CRY and ROC15 and the lifetime distributions of *Cra*CRY:ROC15 double overexpressing control. a. Our results of expression cycles of doubly transfected *Cra*CRY-mCitrine and ROC15-mCherry monitored by fluorescence imaging. C. reinhardtii cells were grown under a LD12:12 cycle (L and D are short for Lit and Dark respectively, and LD means the Lit and Dark alternating cycles) and imaged at the indicated time points. The bar below filled with black from LD14 to LD22 means cells incubated in dark condition, while unfilled part means in lit condition. The number following LD means the time point during the 24-hour cycle (unit: hour). The asterisk indicates the beginning of the next light period at LD02. The 514-nm laser (Fianium ultrafast fiber laser SC-400-4-PP) was used to excite mCherry and mCitrine at the same time. The mCherry and mCitrine fluorescence signal were divided by a 593 nm dichroic (Semrock) and filtered by bandpass filters (BP705/100 for mCherry, Chroma; BP510-555 for mCitrine, Edmund Optics). All the fluorescent photons were collected using a high-speed single-photon counting module (Becker & Hickl HPM-100-40). **b.** Fluorescent images under the dark (left) or lit (right) condition. **c.** Grey and blue lines indicate the lifetime distribution for each image under the dark and lit conditions, respectively. The green arrow points to the characteristic lifetime component of mCitrine signals, used to indicate whether an interaction has occurred (Figure 3, g).

Figure S10 SAXS data and the protein models for dimeric PHR/*Cra*CRY or protein complexes.

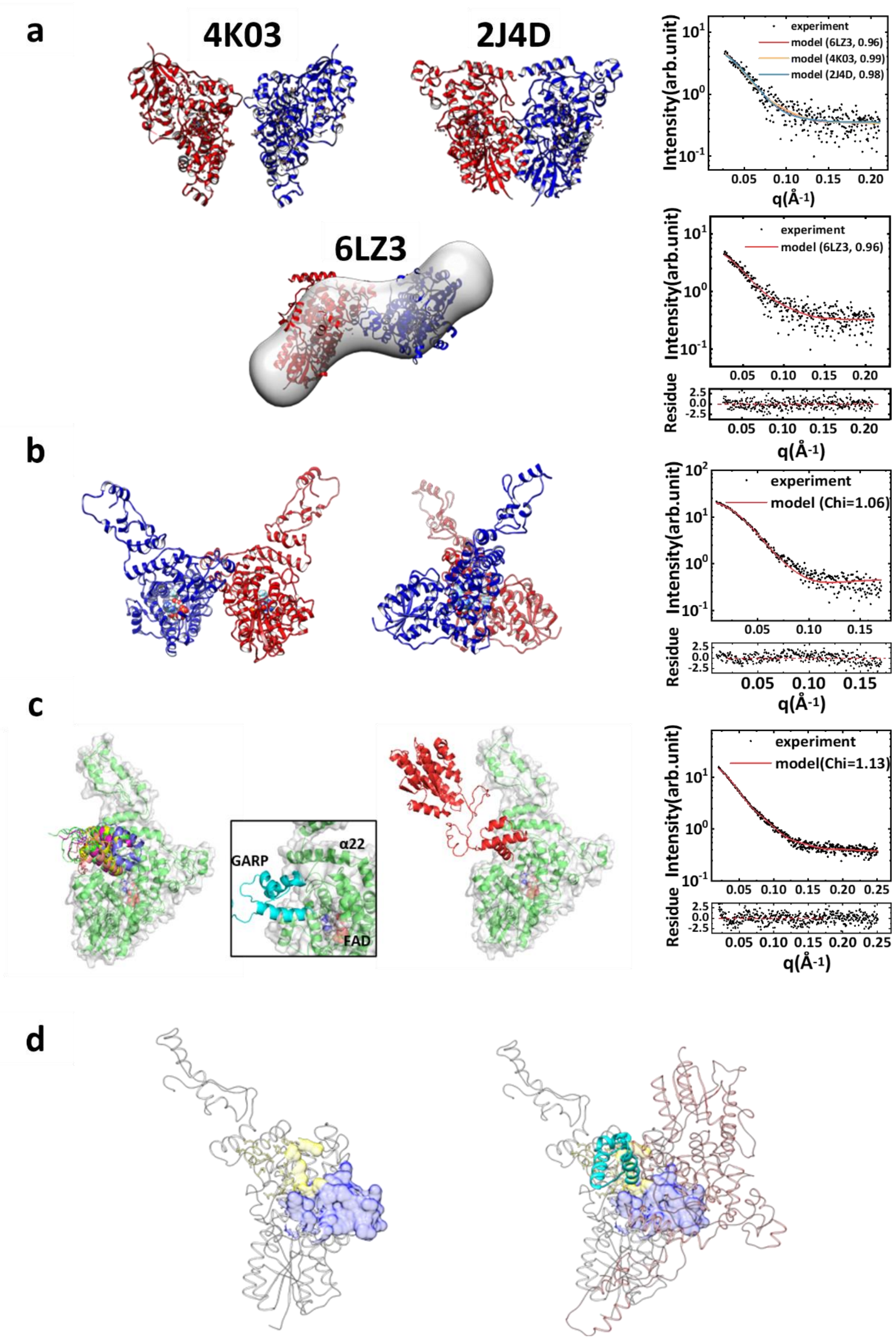

Fig. S10 SAXS data and the protein models for dimeric PHR/*Cra*CRY or protein complexes. **a.** Predicted PHR dimer structure and its theoretical profile fitted to SAXS data. Three models were constructed based on crystal packing and Cryo-EM model templates (the PDB codes are indicated next to the dimer model). The head-to-head dimer model fits to the low-resolution envelope derived from SAXS data (shown as the transparent envelope). The fitting results are shown on the right panels. The residual plot for the chosen model that is based on the Cryo-EM dimer is also sown (right panel, bottom). **b.** Predicted *Cra*CRY dimer structure and its theoretical profile fitted to SAXS data. **c.** The computational docking results of ROC15-GARP on *Cra*CRY. The predicted docking position is near the FAD (left); The model with the highest docking score (middle); the constructed complex model (right) based on the best docking model (the GST-GARP and *Cra*CRY are shown in red and blue cartoon representations) and its fitting to SAXS data. **d.** The ROC15-GARP domain binding region and the *Cra*CRY dimerization interface. The binding residues to GARP domain of ROC15 are shown in stick representation colored in yellow, and the dimerization interface is shown in surface representation: the overlapped region is shown as yellow surface, and the rest of the dimerization interface is shown in blue color. The middle panel shows interface with the inclusion of the GARP and the other *Cra*CRY monomer. The DNA binding motifs (HHLARH [H356-H361] and SQYFR-Y [S409-Y415]) are shown as spheres in green and gold colors in the right panel.

Figure S11 Blue light dependent dimerization, photoreduction and interaction of *Cra*CRY with TCEP or DTT.

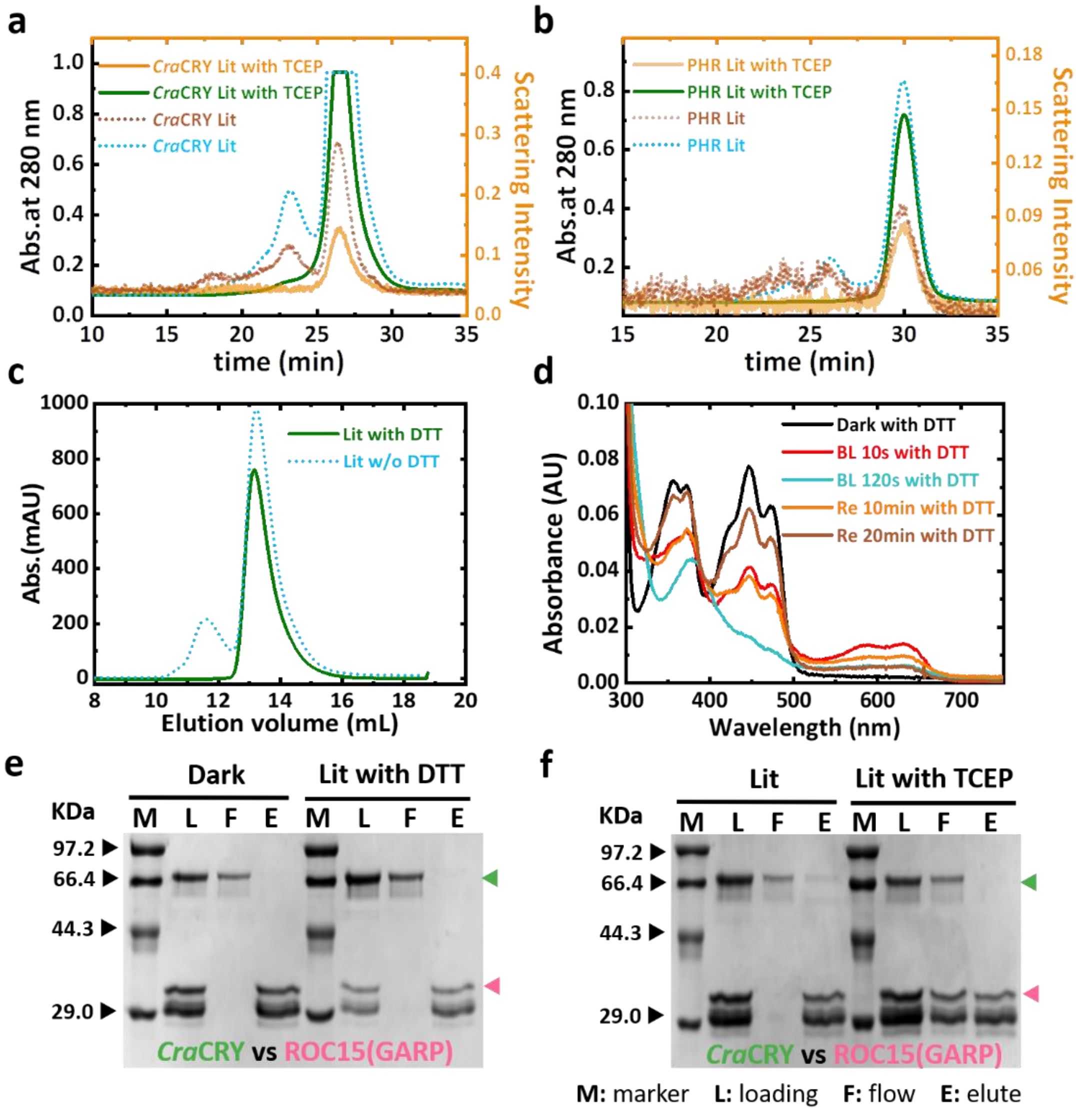

Fig. S11 Blue light dependent dimerization, photoreduction and interaction of *Cra*CRY with TCEP or DTT. a. SEC (green solid) and scattering intensity (orange-yellow solid) curves of *Cra*CRY upon blue-light illumination with 10 mM TCEP. While, the dotted lines are SEC (cyan) and scattering intensity (brown) curves of *Cra*CRY upon blue-light illumination without TCEP. **b.** SEC (green solid) and scattering intensity (orange-yellow solid) curves of PHR domain under dark and lit conditions with 10 mM TCEP. While, the dotted lines are SEC (cyan) and scattering intensity (brown) curves of PHR domain blue-light illumination without TCEP. **c.** SEC (green solid) curve of *Cra*CRY blue-light illumination with 50 mM DTT. While, the cyan dotted line is without DTT. **d.** Photoreduction of purified *Cra*CRY with 10 mM DTT. These absorption spectrums were monitored by UV-VIS spectrometer. Before blue-

light illumination, the *Cra*CRY proteins stay at FAD oxidized state (black line, Dark with DTT). With 10s blue-light illumination, the protein will be partially reduced to FADH state (red line, BL 10s with DTT). And then, *Cra*CRY is further reduced to FAD- state after 120 s blue-light illumination (cyan line, BL 120s with DTT). This process is partly reversible. Part of the reduced protein will return to oxidized state after remaining under dark conditions for 20 min (brown). **e-f.** Pull-down molecular complex analyzed using SDS-PAGE. Green arrows mark the *Cra*CRY(wt). Pink arrows mark the band of GST-ROC15(GARP). The images show that *Cra*CRY(wt) can not be eluted from the GST column under both dark and lit conditions in the presence of DTT/TCEP. It is worth noting, since the blue-light response in absorption spectrum of *Cra*CRY(wt) also changed within DTT/TCEP. The negative interaction results here due to the different spectral response of FAD cannot be ruled out.

Figure S12 Measurement of Förster Radius (R_0) of FRET Dye Pair Atto 550 /Atto 647 in *Cra*CRY buffer.

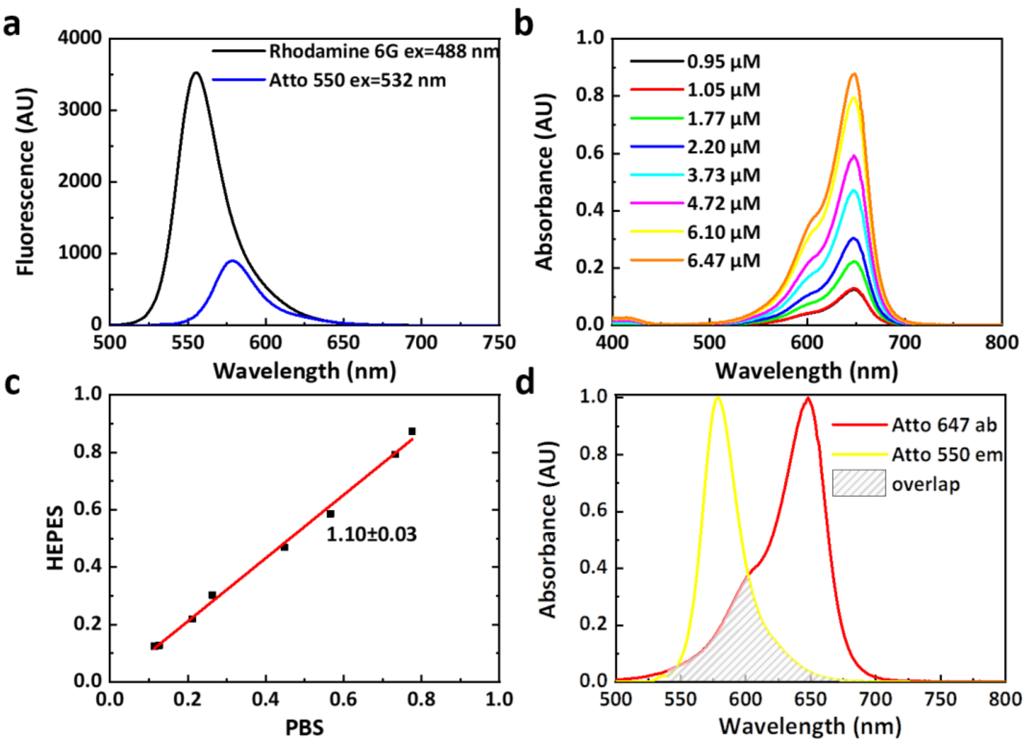

Fig. S12 Measurement of Förster Radius (R_0) of FRET Dye Pair Atto 550/Atto 647 in *Cra*CRY buffer. a. With Rhodamine 6G in water as reference, the quantum yield (QY) of Atto 550 is 0.745 ± 0.002[3]. **b.** The absorption spectra of a series of dilutions of Atto 647 in *Cra*CRY buffer. **c.** Extinction coefficient (EC) of Atto 647 is 132000 $M^{-1}cm^{-1}$ which result from EC of Atto 647 is 120000 $M^{-1}cm^{-1}$ in PBS and absorbance ratio of Atto 647 in *Cra*CRY buffer to PBS buffer is 1.10 ± 0.03. **d.** The overlap of Atto 550 fluorescence and Atto 647 absorption spectra. Finally, the R_0 of Atto550/Atto 647 is calculated as 63.0 ± 0.3 Å from the overlap of Atto 550 fluorescence and Atto 647 absorption spectra, the quantum yield of Atto 550 and the extinction coefficient of Atto 647.

Figure S13 SDS-PAGE images of *Cra*CRY(wt) and FRET-construct during protein expression and purification process.

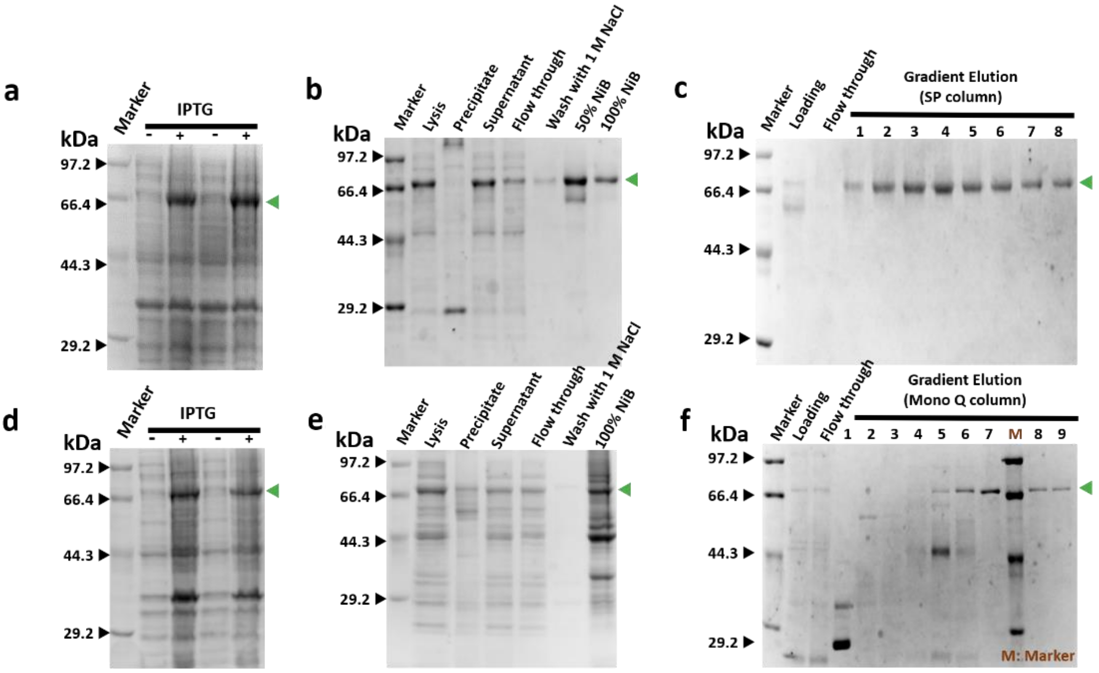

Fig. S13 SDS-PAGE images of *Cra*CRY(wt) and FRET-construct during protein expression and purification process. a. Expression of *Cra*CRY(wt) without (-) /with (+) IPTG induction. The green triangle indicates where the protein should appear. **b.** *Cra*CRY(wt) purified through Ni affinity chromatography. **c.** *Cra*CRY(wt) purified through SP column by gradient elution. **d.** Expression of FRET-construct without (-) /with (+) IPTG induction. The green triangle indicates where the protein should appear. **e.** FRET-construct purified through Ni affinity chromatography. The detergent in lysis buffer was changed to 0.2% CHAPS compared with *Cra*CRY(wt). **f.** FRET-construct purified through Mono Q column by gradient elution. The elution bands of Mono Q column from column 7 to 9 look purer then 100% NiB elution of Ni affinity column.

Figure S14 The constructs of ROC15(GARP) and PHR domain used in the methods.

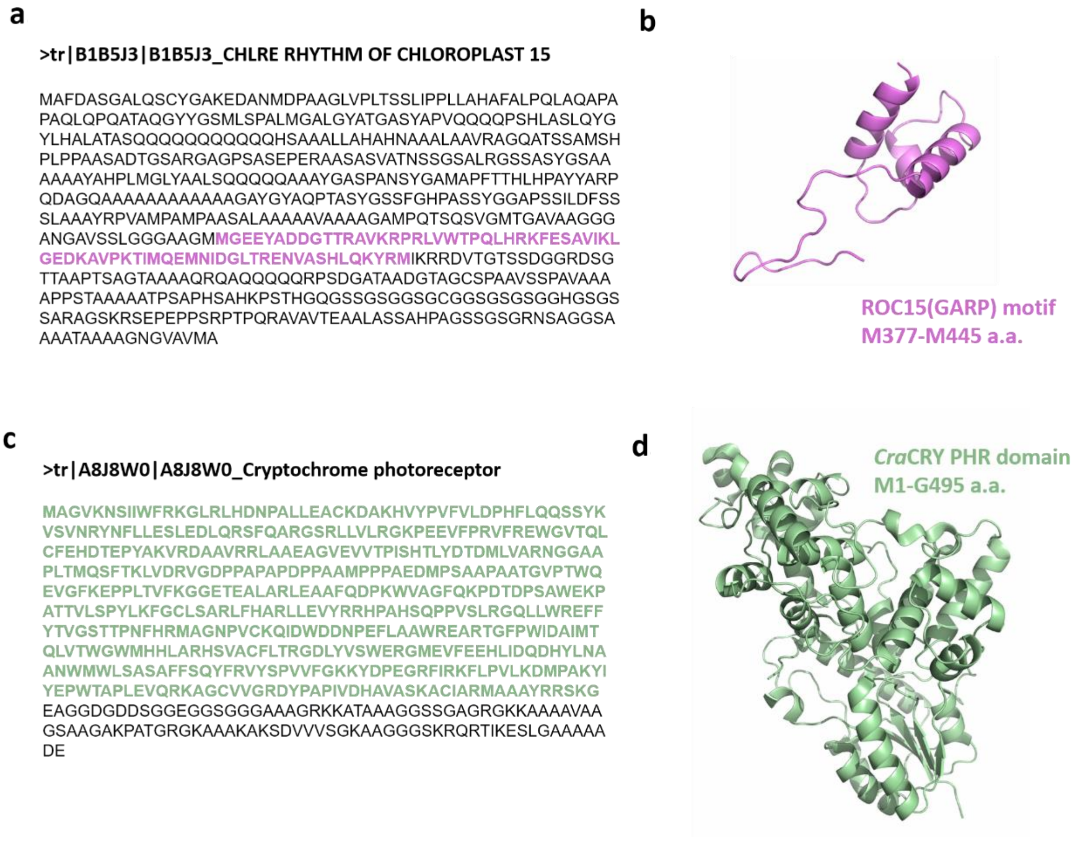

Fig. S14 The constructs of ROC15(GARP) and PHR domain used in the methods. **a.** The amino acid sequence of full-length ROC15. The thick pink part represents the GARP motif (M377-M445 a.a.) in ROC15. **b.** The cartoon structure of ROC15(GARP) motif used in this paper. This structure was predicted by Raptor-X server. **c.** The amino acid sequence of full-length *Cra*CRY. The thick green part represents the PHR domain (M1-G495 a.a.) in *Cra*CRY. **d.** The cartoon structure of PHR domain used in this paper. This structure was downloaded from PDB bank (ID: 5zm0).

Movie S1 Refinement of *Cra*CRY protein by SAXS-driven MD simulations (dark). This movie is generated from SAXS-driven MD Simulation, starting with the model predicted by our data-guided modeling program. The representative model is shown in gray, while the orange cartoon model shows the progression of structural refinement against monomer *Cra*CRY SAXS data in dark state.

Please see the video file named 'Supplementary Movie 1.mpg'.

Movie S2 Refinement of *Cra*CRY protein by SAXS-driven MD simulations (lit). Same as Movie 1, except that the representative model is shown in blue color, while the orange cartoon model shows the progression of structural refinement against lit-state *Cra*CRY SAXS data.

Please see the video file named 'Supplementary Movie 2.mpg'.

Movie S3 Refinement of *Cra*CRY protein by SAXS-driven MD simulations (trRosetta-lit). The starting model is predicted by trRosetta. The blue cartoon model shows the same representative model as in Movie 2, the refinement process is shown as the trace of protein backbone. The refinement target SAXS data is from the full-length *Cra*CRY in lit-state.

Please see the video file named 'Supplementary Movie 3.mpg'.

Movie S4 *Cra*CRY protein dynamics with conventional equilibrium MD simulations. This MD simulation started with our predicted model (same as Movies S1 and S2), except that the SAXS-data was not applied as restraints. This simulation trajectory shows that the CTE has strong tendency to bind to PHR domain, converging to a conformation similar to the trRosetta predicted structure, which is shown as the backbone trace colored in cyan (see also Movie S3). However, the modes observed in this simulation are not consistent with SAXS-data.

Please see the video file named 'Supplementary Movie 4.mpg'.

Supplementary References

1. X. Sun, D. Montiel, H. Li, and H. Yang, 'Plug-and-Go' Strategy To Manipulate Streptavidin Valencies, Bioconjug. *Chem.*, **25**, 1375–1380 (2014).
2. Li, P., Dai, Y., Seeger, M. & Tan, Y.-W. Quantifying Intramolecular Protein Conformational Dynamics Under Lipid Interaction Using smFRET and FCCS. *Methods Mol Biol.* **1860**, 345–359 (2019).
3. Douglas Magde, Roger Wong and Paul G. Seybold, Fluorescence Quantum Yields and Their Relation to Lifetimes of Rhodamine 6G and Fluorescein in Nine Solvents: Improved Absolute Standards for Quantum Yields, *Photochemistry and Photobiology*, **75**, 327–334 (2002).
