# Supplementary material for: Direct experimental observation of blue-light-induced conformational change and intermolecular interactions of cryptochrome
Source: Commun Biol. 2022 Oct 18;5:1103. doi: 10.1038/s42003-022-04054-9 (PMC9579160; doi:10.1038/s42003-022-04054-9)
Supplement: Supplementary file 3 — Description of Additional Supplementary Files [file 42003_2022_4054_MOESM3_ESM.pdf]

Description of Additional Supplementary Files

File name: Supplementary Data 1
Description: PDB files for protein constructs in the manuscript.

File name: Supplementary Data 2
Description: The source data (.xlsx) behind the graphs in the paper.

File name: Supplementary Movie S1
Description: Refinement of CraCRY protein by SAXS-driven MD simulations (dark).

File name: Supplementary Movie S2
Description: Refinement of CraCRY protein by SAXS-driven MD simulations (lit).

File name: Supplementary Movie S3
Description: Refinement of CraCRY protein by SAXS-driven MD simulations (trRosetta-lit).

File name: Supplementary Movie S4
Description: CraCRY protein dynamics with conventional equilibrium MD simulations.
