# Supplementary material for: Direct experimental observation of blue-light-induced conformational change and intermolecular interactions of cryptochrome
Source: Commun Biol. 2022 Oct 18;5:1103. doi: 10.1038/s42003-022-04054-9 (PMC9579160; doi:10.1038/s42003-022-04054-9)
Supplement: Supplementary file 9 — Reporting Summary [file 42003_2022_4054_MOESM9_ESM.pdf]

nature portfolio

Corresponding author(s): Yan-Wen Tan, Haiguang Liu

Last updated by author(s): Sep 18, 2022

Reporting Summary

Nature Portfolio wishes to improve the reproducibility of the work that we publish. This form provides structure for consistency and transparency in reporting. For further information on Nature Portfolio policies, see our Editorial Policies and the Editorial Policy Checklist.

Statistics

For all statistical analyses, confirm that the following items are present in the figure legend, table legend, main text, or Methods section.

n/a	Confirmed	
☐	☒	The exact sample size (n) for each experimental group/condition, given as a discrete number and unit of measurement
☐	☒	A statement on whether measurements were taken from distinct samples or whether the same sample was measured repeatedly
☐	☒	The statistical test(s) used AND whether they are one- or two-sided<br>*Only common tests should be described solely by name; describe more complex techniques in the Methods section.*
☐	☒	A description of all covariates tested
☐	☒	A description of any assumptions or corrections, such as tests of normality and adjustment for multiple comparisons
☐	☒	A full description of the statistical parameters including central tendency (e.g. means) or other basic estimates (e.g. regression coefficient) AND variation (e.g. standard deviation) or associated estimates of uncertainty (e.g. confidence intervals)
☐	☒	For null hypothesis testing, the test statistic (e.g. F, t, r) with confidence intervals, effect sizes, degrees of freedom and P value noted<br>*Give P values as exact values whenever suitable.*
☒	☐	For Bayesian analysis, information on the choice of priors and Markov chain Monte Carlo settings
☐	☒	For hierarchical and complex designs, identification of the appropriate level for tests and full reporting of outcomes
☒	☐	Estimates of effect sizes (e.g. Cohen's d, Pearson's r), indicating how they were calculated

Our web collection on statistics for biologists contains articles on many of the points above.

Software and code

Policy information about availability of computer code

Data collection	The softwares for controlling EMCCD (Andor, DU897E), ultrafast fiber Laser (Fianium, SC-400-4-PP) and FLIM microscope (Becker & Hickl, SPCM) are from the original factories.
Data analysis	Single-molecular data were analyzed by ImageJ and Matlab. Fluorescence lifetime analysis were finished by the original software (Becker & Hickl, SPCimage). Lifetime image drawing was using by Matlab. SAXS-driven MD simulation was using the modified source code of GROMACS 4.6.5. VMD and in-house Python scripts were employed to visualize and analyze the output of the MD simulations, respectively. The full-length structure of CraCRY was predicted using a template-based modeling protocol and the global optimization method called conformational space annealing (CSA).

For manuscripts utilizing custom algorithms or software that are central to the research but not yet described in published literature, software must be made available to editors and reviewers. We strongly encourage code deposition in a community repository (e.g. GitHub). See the Nature Portfolio guidelines for submitting code & software for further information.

Data

Policy information about availability of data

All manuscripts must include a data availability statement. This statement should provide the following information, where applicable:

- Accession codes, unique identifiers, or web links for publicly available datasets
- A description of any restrictions on data availability
- For clinical datasets or third party data, please ensure that the statement adheres to our policy

These data are already included in the supplementary tables.

Human research participants

Policy information about studies involving human research participants and Sex and Gender in Research.

Reporting on sex and gender	Our studies did not involve any animals and human research participants.
Population characteristics	Our studies did not involve any animals and human research participants.
Recruitment	Our studies did not involve any animals and human research participants.
Ethics oversight	The author declares no competing interests.

Note that full information on the approval of the study protocol must also be provided in the manuscript.

Field-specific reporting

Please select the one below that is the best fit for your research. If you are not sure, read the appropriate sections before making your selection.

☒ Life sciences ☐ Behavioural & social sciences ☐ Ecological, evolutionary & environmental sciences

For a reference copy of the document with all sections, see nature.com/documents/nr-reporting-summary-flat.pdf

Life sciences study design

All studies must disclose on these points even when the disclosure is negative.

Sample size	We repeated the parallel experiments as many times as possible until the statistics no longer changed significantly.
Data exclusions	Data with very low signal-to-noise ratios or anomalous curves with three or fewer occurrenches were excluded.
Replication	More then three parallel experiments repeated for each experimental findings.
Randomization	All the parallel experiments were performed on different date and using the freshly prepared protein specimen.
Blinding	Data screening was done automatically by algorithm based on specific criterion.

Reporting for specific materials, systems and methods

We require information from authors about some types of materials, experimental systems and methods used in many studies. Here, indicate whether each material, system or method listed is relevant to your study. If you are not sure if a list item applies to your research, read the appropriate section before selecting a response.

Materials & experimental systems

n/a	Involved in the study	
☐	☒	Antibodies
☐	☒	Eukaryotic cell lines
☒	☐	Palaeontology and archaeology
☒	☐	Animals and other organisms
☒	☐	Clinical data
☒	☐	Dual use research of concern

Methods

n/a	Involved in the study	
☒	☐	ChIP-seq
☒	☐	Flow cytometry
☒	☐	MRI-based neuroimaging

Antibodies

Antibodies used	The clone code of his-tag antibody for western blot test on the wild-type CraCRY is AD1.1.10.
Validation	AD1.1.10. was bought from Beyotime (China). Details are shown at website https://www.beyotime.com/product/AH367.htm.

Eukaryotic cell lines

Policy information about cell lines and Sex and Gender in Research

Cell line source(s)	C. reinhardtii (UVM11) cells
Authentication	We got the UVM11 cells from Prof. Guoliang Xu and Dr. Jianhuang Xue in Shanghai Institutes for Biological Science (SIBS), and could not authenticate by ourselves subject to technological conditions.
Mycoplasma contamination	We did not test the mycoplasma contamination. However, no abnormality of cell morphology and growth rate was found.
Commonly misidentified lines (See ICLAC register)	UVM4
